# Topoclimate effect on treeline elevation depends on the regional framework: A contrast between Southern Alps (New Zealand) and Apennines (Italy) forests

**DOI:** 10.1002/ece3.9733

**Published:** 2023-01-16

**Authors:** Angelo Rita, Antonio Saracino, Ellen Cieraad, Luigi Saulino, Maurizio Zotti, Mohamed Idbella, Carlo De Stefano, Valentina Mogavero, Emilia Allevato, Giuliano Bonanomi

**Affiliations:** ^1^ Dipartimento di Agraria Università degli Studi di Napoli Federico II Portici Italy; ^2^ Research & Innovation Centre Nelson Marlborough Institute of Technology Nelson New Zealand; ^3^ Laboratory of Biosciences, Faculty of Sciences and Techniques Hassan II University Casablanca Morocco; ^4^ Task Force on Microbiome Studies Università degli Studi di Napoli Federico II Naples Italy

**Keywords:** anthropogenic disturbance, land use, mean annual temperature, Topoclimate, Treeline isotherm

## Abstract

Deciphering the spatial patterns of alpine treelines is critical for understanding the ecosystem processes involved in the persistence of tree species and their altitudinal limit. Treelines are thought to be controlled by temperature, and other environmental variables but they have rarely been investigated in regions with different land‐use change legacies. Here, we systematically investigated treeline elevation in the Apennines (Italy) and Southern Alps (New Zealand) with contrasting human history but similar biogeographic trajectories, intending to identify distinct drivers that affect their current elevation and highlight their respective peculiarities. Over 3622 km of Apennines, treeline elevation was assessed in 302 mountain peaks and in 294 peaks along 4504 km of Southern Alps. The major difference between the Southern Alps and Apennines treeline limit is associated with their mountain aspects. In the Southern Alps, the scarcely anthropized *Nothofagus* treeline elevation was higher on the warmer equator‐facing slopes than on the pole‐facing ones. Contrary to what would be expected based on temperature limitation, the elevation of *Fagus sylvatica* treelines in the Apennines was higher on colder, pole‐facing slopes than on human‐shaped equator‐facing, warmer mountainsides. Pervasive positive correlations were found between treeline elevation and temperature in the Southern Alps but not in the Apennines. While the position of the *Fagus* and *Nothofagus* treelines converge on similar isotherms of annual average temperature, a striking isothermal difference between the temperatures of the hottest month on which the two taxonomic groups grow exists. We conclude that actual treeline elevation reflects the ecological processes driven by a combination of local‐scale topoclimatic conditions, and human disturbance legacy. Predicting dynamic processes affecting current and future alpine treeline position requires further insight into the modulating influences that are currently understood at a regional scale.

## Graphical abstract

The major difference between the Southern Alps and Apennines treeline limit is associated with their mountain aspects. Contrary to what would be expected based on temperature limitation, the elevation of *F. sylvatica* treelines in the Apennines was higher on colder, pole‐facing slopes than on equator‐facing, warmer mountainsides. While the position of the *Fagus* and *Nothofagus* treelines converge on similar isotherms of annual average temperature, a striking isothermal difference between the treelines concerning the temperatures of the hottest month on which the two taxonomic groups grow exists. We conclude that actual treeline elevation reflects the ecological processes driven by a combination of local‐scale topoclimatic conditions, and human disturbance legacy.

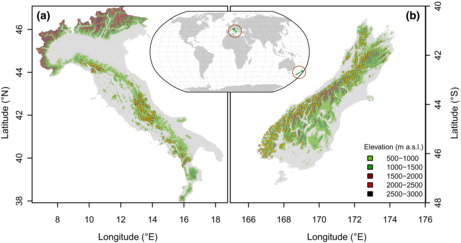

## 1 INTRODUCTION

The alpine treeline is part of a clear‐cut ecotone that represents a marked ecological boundary between the upper elevation limit of closed‐canopy forests and the alpine vegetation (Körner, 2012). The elevation of treelines spans from a few hundred meters above sea level in boreal areas to more than 4000 m a.s.l. in the Himalayas and at tropical latitudes (Kessler et al., 2014; Miehe et al., 2007). Several studies identifying the role of potential drivers that control alpine treeline elevation agree that temperature is the most important factor constraining tree growth and development at high altitudes (e.g., Harsch et al., 2009). Although mountain forests may be exposed to severe frost damage (Allevato et al., 2019; Zohner et al., 2020), the most supported hypothesis explaining the lack of trees above a certain altitude proposes that low temperatures limit cambial (e.g., Li et al., 2017) and meristematic (i.e., shoot length) tissue production, but not photosynthesis (e.g., Hoch & Körner, 2012, but see Fajardo & Piper, 2017).

Despite significant research efforts relating to treelines around the world, some uncertainties still remain concerning the nonlinear response of global treelines to recent climate warming. While there is consensus that 20th‐century macroclimate warming has played a pivotal role in triggering a recent upward expansion of alpine treelines (e.g., Kullman, 2007; Liang et al., 2016), the feedback of emerging contingent fine‐scale factors is still a matter of debate (e.g., Camarero et al., 2021; Körner, 2021a), as are detailed other local mechanisms that may contribute to treeline position or limit the ability of trees to respond to climate change (Harsch & Bader, 2011). For example, topographical conditions coupled with local (e.g., extreme winds, snow avalanches, rockfall) and anthropogenic (e.g., fires, grazing by livestock) disturbances can significantly depress treeline elevation compared to their climatic potential (Ameztegui et al., 2016; Bonanomi et al., 2020; Harsch et al., 2012; Holtmeier & Broll, 2020). This makes understanding finer‐scale variation in drivers of treeline difficult. At a regional scale, tree spatial pattern slows down the impact of the warming climate and is considered a crucial factor in shaping treeline position (Bader et al., 2021; Sigdel et al., 2020). Similarly, precipitation patterns along a topographic gradient can complicate temperature‐treeline relationships. For example, treelines are depressed if the soil moisture falls below a critical threshold even when temperatures are warm enough, or if the interaction between high temperature and low precipitation causes drought stress (D'Arrigo et al., 2009). Moreover, at local scales, aspect and steepness critically determine the potential amount of solar radiation reaching vegetation and ground surface (Monteith & Unsworth, 2013). Mountainside aspects and microtopography can thus also affect treeline elevation through local, but dramatic, variations in temperature and soil moisture (Körner, 2021b), and thus near‐ground temperature. At much larger scales, at mid and high latitudes, the pole‐facing slopes receive much less solar radiation than equator‐facing slopes with important consequences for heat balance. It would be expected therefore that treeline elevation would be higher on warmer equator‐facing slopes, i.e., south‐facing and north‐facing in the northern and southern hemisphere, respectively, than on colder pole‐facing ones, i.e., north‐facing and south‐facing in the northern and southern hemisphere, respectively.

The steepness of mountain slopes drives treeline elevation. While on extremely steep slopes, mechanical forces and erosion tend to limit tree establishment and impair growth, on moderate slopes snow accumulation may lead to an increase in avalanches that can locally reduce treeline elevation (Case & Duncan, 2014; Cullen et al., 2001; Holtmeier & Broll, 2020). Snow accumulation on gentler slopes promotes protection of outpost treeline groups against severe winter conditions while being more accessible for logging, and grazing compared to steeper areas. In this regard, studies in the European mountains such as Alps, Pyrenees, and the Apennines found that abrupt transitions from forests to alpine prairies correspond to less steep slope areas (e.g., Ameztegui et al., 2016; Bonanomi et al., 2018, 2020; Gehrig‐Fasel et al., 2007). Paleoclimatic records further corroborate that high‐elevation European secondary prairies have been expanded to the detriment of forests by extensive burning, logging, and subsequent pastoral uses (Feurdean et al., 2016; Gehrig‐Fasel et al., 2007; Morales‐Molino et al., 2021; Tinner et al., 1996). This is in line with a contemporary study of Batllori and Gutiérrez (2008) suggesting that treeline dynamics of *Pinus uncinata* in the Pyrenees have been widely affected by local anthropogenic activities. In addition, Bonanomi et al. (2018) reported that the present Italian Apennines' *Fagus sylvatica* treeline is several hundred meters below its climatic potential because of widespread land‐use change, as confirmed from several paleoecological findings (e.g., Benatti et al., 2019; Brown et al., 2013). Therefore, any hypotheses of treeline dynamics, at local than global scales, in response to climate warming should at least consider the complex interaction among thermal requirements, physiological stressors, and disturbance regime, especially in mountains with a long history of human exploitation (Ameztegui et al., 2016).

In a global treeline comparison, Körner and Paulsen (2004) revealed surprisingly similar mean temperatures during the local growing seasons, independent of the climatic zone, the length of the growing season, the seasonal amplitude of temperatures, and the extreme winter climate. Irrespective of species occurrence, high‐altitude climatic treelines are associated with a seasonal mean ground temperature of 6.7°C ± 0.8 SD, a rather narrow range that sets a physiological threshold to trees linked to low temperatures (Körner & Paulsen, 2004). The authors noted that a number of Mediterranean (*Fagus* spp.) and temperate South Hemisphere treelines (*Nothofagus* spp., also known as southern beech) in New Zealand and Chile, and the native treeline in Hawaii (*Metrosideros* spp.) did not match the mean global treeline isotherm lying at substantially higher isotherms and they proposed that these represent genus‐specific boundaries rather than boundaries of the life‐form tree. However, more recently, detailed local studies have shown that, at least in New Zealand and southern Patagonia, treelines fall within the global isotherm range of the first study (Cieraad et al., 2014; Hertel et al., 2008). Instead, the thermal limit of *Fagus* treelines along the Apennines is thought to be located well below the potential limit (i.e., “too warm”).

Although annual mean temperatures are widely recognized as the main driver of treeline elevation, we support the hypothesis that its effect on the current treeline elevation could be mediated by other factors such as past anthropogenic activity. To test our hypothesis, in this work we contrasted a human‐shaped Italian Apennines treelines with a relatively undisturbed New Zealand Southern Alps one. The New Zealand Southern Alps and the Italian Apennines are both axial mountain ranges, crossing the Italian peninsula and the New Zealand South Island, respectively, roughly from north to south, under the bioclimatic influence of weather systems from the sea. While in different Hemispheres, both lie at similar latitudes. However, the mountain ranges have strongly different land‐use histories. The Apennines have a millennial history of well‐attested intense human disturbance from the Bronze Age through the Roman period (Barker, 1995) until the mid‐1900s. Such a disturbance included extensive forest logging up to the subalpine belt for charcoal production, firewood, grazing, and wood exploitation for the newborn Italian railway network (Malanima, 2006). In the last century, socioeconomic changes (e.g., mountain land abandonment) and establishment of protected areas have resulted in a reduction in direct human disturbance (Corona, 2015). Conversely, New Zealand forests have been little affected by human activities, and, to date, the population density is approximately 11 times lower than in Italy (World Bank, 2022). Before arrival of Māori about 700–800 years ago, forest covered much of the New Zealand landscape, and since the second half of the 1800s, when Europeans were a small part of the total population, fires mainly affected lowland and rain‐shadow mountains, largely leaving *Nothofagus* forests – including forest limits – intact (McGlone & Wilmshurst, 1999; Wardle, 2008).

Although some climatic and geographic patterns in the Italian and New Zealand treeline ecotone have been recognized (e.g., Bonanomi et al., 2018; Case & Buckley, 2015; Wardle, 2008), the factors characterizing these differential patterns are still unknown or have not been systematically described. We suspect that, compared to the *Nothofagus* treelines of New Zealand's Southern Alps, the legacy of past human land use has affected the actual position, and consequently the thermal limit, of *Fagus* treelines in the Italian Apennines.

In this context, the general aim of this study is to systematically investigate treeline elevation of *F. sylvatica* and *Nothofagus* spp.in two subregions (Apennines and Southern Alps, respectively) with contrasting human history but relatively similar biogeographic trajectories, intending to identify the common determinants that affect their present‐day elevation and highlight their respective peculiarities. In detail, we tested the following specific hypotheses: (i) *Nothofagus* treeline elevation is relatively higher on warmer mountain slopes in the Southern Alps in contrast to *Fagus* in the Apennines; (ii) compared to Apennines, the Southern Alps treeline elevation would be under macroclimatic control because of the relative limited anthropogenic disturbance. To achieve these aims, we measured treeline elevation on all aspects of 596 peaks along the two mountain ranges, providing an accurate localization of treeline occurrence, and assessed the contribution of climatic and topographic factors in affecting treeline elevation.

## 2 MATERIALS AND METHODS

### 2.1 Study areas

**FIGURE 1:**
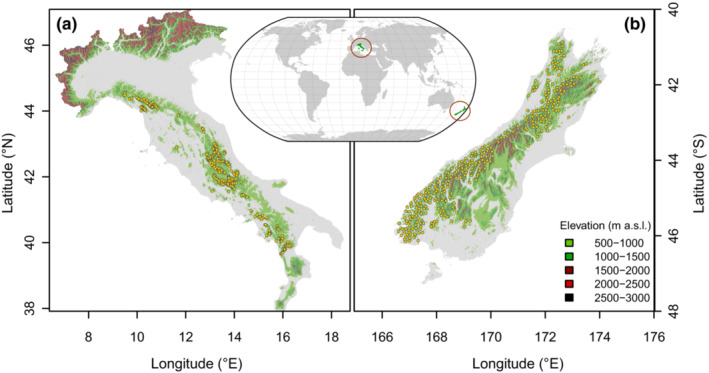
Location of Apennines Mountain chain with the 302 selected peaks (a) and southern Alps with the 294 studied peaks (b). Peaks are indicated with yellow dots in both panels.

Treeline elevation assessments were conducted for the Apennines in Italy and the Southern Alps in New Zealand (Figure 1). The Apennines is a mountain chain of ~1200 km length that follows the length of the Italian peninsula between 38° and 44° of latitude N and surrounded by Mediterranean sea. The mountain range is under the influence of a warm‐summer Mediterranean and temperate oceanic climate, according to the Köppen‐Geiger classification system. The Apennines include 261 major peaks above 2000 m a.s.l. Geologically, the northern Apennines largely consist of arenaceous‐pelitic flysch substrate, while the central and southern Apennines (excluding Calabrian‐Peloritan arc) largely consist of limestone substrate (Bosellini, 2017). *Fagus sylvatica* is the most common deciduous tree in the mountain and subalpine vegetation belts and typically forms monospecific stands at the treeline. Sporadically, *F. sylvatica* at treeline coexists with conifer species, including *Picea abies* in the northern Apennines (Magini, 1972), and *Pinus nigra* (Piermattei et al., 2012), and *Pinus heldreichii* subsp. *leucodermis* (Todaro et al., 2007) in central and southern Apennines, respectively.

The Southern Alps represent the highest range in Australasia extending latitudinally along the South Island (Te Waipounamu) from 46° to 40°S for ~650 km and surrounded by the Pacific Ocean. The Southern Alps are higher than the Apennines, they boast ~360 glaciers, and contain 16 peaks above 3000 m a.s.l. (including the highest peak, Mount Cook, at 3754 m a.s.l.). Geologically, the Southern Alps are mainly composed of hard sandstone and mudstone, usually indicated as “greywacke”. In the southern and western parts, the Southern Alps are also formed by schist. Along the Southern Alps, the climate spans from subpolar oceanic to tundra climate according to Köppen‐Geiger climate classification. Several tree species form treelines including several species in the *Nothofagus* clade (Heenan & Smissen, 2013, further referred to as *Nothofagus* spp.). *Nothofagus menziesii* is dominant in the wetter western area, and *Fuscospora cliffortioides* (formerly *Nothofagus solandri* var. *cliffortioides*) is more common in the drier eastern mountains (Cieraad & McGlone, 2014; Cullen et al., 2001). In many western areas, treeline is formed by other species including *Dracophyllum* spp., *Libocedrus bidwillii*, *Halocarpus biforme*, and *Phyllocladus alpinus*. The exotic *Pinus contorta* also forms a treeline in some locations (Cieraad & McGlone, 2014).

### 2.2 Treeline assessment

The Apennines dataset used for this study came from our previously published dataset that includes treeline assessed in mountain peaks with an elevation above 1500 m a.s.l. (Bonanomi et al., 2018). Overall, 302 mountain peaks were included with 3622 km of treelines measured using Google Earth Pro™ images (Google, Inc.; Figure 1). Here, we extended the same methodological approach to the Southern Alps in New Zealand where the treeline was carefully mapped. The treeline elevation was identified by a supervised digitalization of the boundary between forest and grassland on the four aspects of each selected peak (see for example Figure S1). Afterward, the digitized vector lines were measured for their minimum, average, and maximum elevation above sea level. Because of the large number of peaks in the Southern Alps, we randomly selected a subset of peaks in which treelines are evident. First, we generated a 15 km × 15 km square grid covering the entire South Island of New Zealand. Then, we identified all squares with several mountains containing evident treelines, where a single peak for treeline elevation assessment was randomly selected. Using this approach, we selected 294 peaks along the length of the Southern Alps (Figure 1). We did not differentiate between different native treeline species, as this was outside the scope of this study. We excluded sites with the exotic *P. contorta* from treeline assessment, being sometimes planted near or above the pre‐existing treeline. Delineated *Fagus* and *Nothofagus* treelines were further checked for consistency by overlapping them with the Corine Land Cover 2018 dataset (CLC18, available for download at https://sinacloud.isprambiente.it/arcgisina/rest/services/corine_land_cover/) that provides a complete spatial extent of landcover ‘Beech forests’ across Italy, and with the New Zealand Landcover Database (LCDB v5.0, available for download at https://lris.scinfo.org.nz/layer/104400‐lcdb‐v50‐land‐cover‐database‐version‐50‐mainland‐new‐zealand/), which provides a complete spatial representation of landcover ‘Indigenous Forests’ across New Zealand at the ca 1:50,000 scale derived from satellite imagery using GIS software (QGIS v.3.14.15‐Pi).

### 2.3 Climatic, geographic, and topographic variables

To identify variables associated with treeline elevation, we used a combination of climatic, geographic, and topographic variables that are correlated with treeline elevation (Bonanomi et al., 2018; Holtmeier & Broll, 2020). In detail, climatic data (Table S1) were gathered from the database WorldClim 2.0 (http://www.worldclim.org; Hijmans et al., 2005) at a spatial resolution of 1 km^2^. Temperature‐associated variables in the WorldClim represent the average of grid squares adjusted according to their average elevation (derived from the SRTM elevation data 1 km^2^). Following Jobbagy and Jackson (2000) we removed the effect of the grid elevation by scaling mean annual (MAT_adj_), mean monthly (MMT_adj_), and mean quarterly (MQT_adj_) temperature variables at sea level, and then recalculating temperature variables at treeline elevation (i.e., MAT_tl_, MMT_tl_, MQT_tl_, respectively) using an adiabatic lapse rate of 0.006°C m^−1^. Using a thermal lapse rate simplifies the calculation of adjusted, and therefore comparable, temperatures, and is considered suitable for temperate and cold regions (Barry, 2008) although we recognize lapse rates may be more variable under the cool temperate oceanic climates (e.g., Cieraad & McGlone, 2014). This lapse rate approach was applied to mean annual temperature, mean monthly temperature, and mean quarterly temperatures. For rainfall, we used non‐scaled monthly, seasonal, and annual precipitation. Concerning geographical and topographic variables, we included latitude, longitude, peak elevation, and slope steepness at the treeline.

### 2.4 Data analysis

For the Apennines and the Southern Alps, minimum, average, and maximum treeline elevations were calculated from the treeline elevation assessment conducted using Google Earth Pro TM images. Then unpaired two‐sample *t*‐test was used to test the null hypothesis (for *p* < .05) that the average treeline elevation of the two mountain regions did not differ per aspect. To avoid confusion due to the opposite solar exposure of northern and southern aspects in the two hemispheres, we refer to cold aspects (i.e., pole‐facing: northern for the Apennines and southern for the Southern Alps) and warm aspects (i.e., equator‐facing: southern for the Apennines and northern for the Southern Alps).

To test the significance of a set of environmental predictors on the treeline elevation, relationships between average treeline elevation and geographical, topographic, and climatic variables adjusted to sea level were quantified using both Spearman's rank correlations and partial least square (PLS) regressions. PLS is a robust statistical approach designed to handle non‐independence or high collinearity among predictors in describing their linear relationships with treeline elevation. The most interesting feature of PLS is that the relationships between predictors and the response variable can be inferred from weights and regression coefficients of individual predictors in the most explanatory components. We implemented PLS regressions using the R package *mdatools* v0.13.0 (Kucheryavskiy, 2020) of R open‐source statistical environment (R Core Team, 2021). Models were built using a calibration set and full cross‐validation. The optimal number of components to be retained in the models was identified by looking at the minimum root mean square error (RMSE) for the cross‐validated predictions. The contribution of individual predictors was evaluated using the variable importance in the projection (VIP, i.e., the strength of influence for each predictor) and the regression coefficients (i.e., the direction of the influence) and corresponding inferential analysis carried out by Jack‐Knifing approach. Prior to the PLS analysis, all variables were square root transformed, centered, and scaled to unit standard deviation to improve the symmetry of the distributions and enable comparability of the coefficients of the predictors measured on different scales.

We tested for nonlinearity of the relationships between the average treeline elevation and geographical variables using Generalized Additive Mixed Models (GAMMs) for both Southern Alps and Apennines including separately latitude and longitude as fixed effects and the mountain mass ID and mountainside aspects and slope as random effects with the *mgcv* (Wood, 2011) R package. The level of complexity (nonlinearity) of model terms has been determined by the estimated degrees of freedom (e.d.f.) of the smoother by using approximate restricted maximum likelihood (REML) method, with low REML values representing the best compromise between model complexity and fit to the observed data. Diagnostic plots, i.e., the distribution of residuals and quantile‐quantile plots, were used to evaluate whether the assumption of a normal distribution was suitable and the goodness‐of‐fit was determined by calculating the conditional coefficient of determination (R^2^) as using the ‘*r.squaredGLMM*’ function in the *MuMIn* (Barton, 2022) package.

For each mountain chain, treeline isotherms were modeled by fitting extreme value distributions to climate data with the *extRemes* v2.0 package (Gilleland & Katz, 2016). In all cases, the *fevd* routine was used to fit a Generalized Extreme Value (GEV) distribution to scaled mean annual temperature at the treeline (MAT_t_, continuous variable), mean temperature of the coldest month (i.e., February and August for Apennines and Southern Alps, respectively), mean temperature of the warmest month (i.e., August and February for Apennines and Southern Alps, respectively). The models were fit using the default maximum likelihood estimation and 95% confidence intervals were estimated using bootstrap resampling technique with 10,000 iterations.

## 3 RESULTS

**FIGURE 2:**
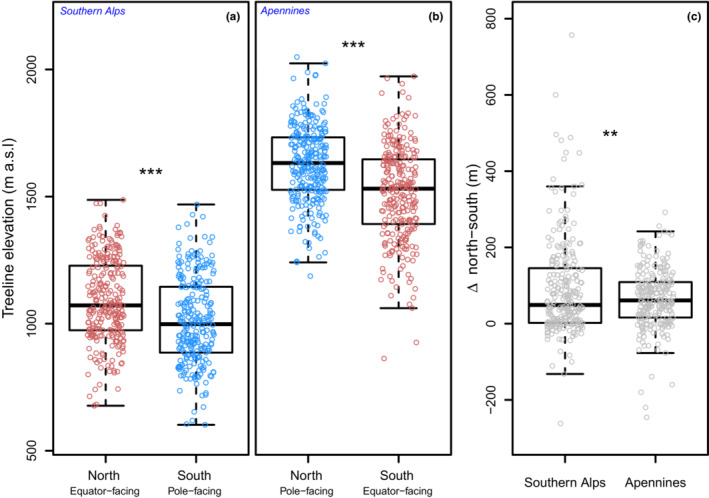
Average treeline elevation in north and south aspects in the southern Alps (a) and Apennines (b). Difference (Δ) of maximum treeline elevation between north and south aspect (c) stars indicate statistical significance (two‐tailed *t*‐test) for between‐group comparisons. ***p < .001, **p < .01.

Overall, 4504 km of treeline on 294 mountain peaks were measured for the Southern Alps which ranged in elevation between 401 and 1586 m a.s.l. The Apennines dataset consisted of 3622 km of treeline on 302 peaks, ranging from 814 to 2141 m a.s.l. The mean treeline elevation was 1060 ± 173 m a.s.l. in the Southern Alps, much lower than in the Apennines (1589 ± 178 m a.s.l.). The treeline elevation was, on average, 78 m higher on warm than on cold slopes in the Southern Alps (*t*_(504.84)_ = 5.4873, *p* < .001; Figure 2). In contrast, treeline elevation in the Apennines was, on average, 114 m lower on warm slopes than on colder ones (*t*_(511.34)_ = 7.7525, *p* < .001; Figure 2). Comparing all mountainside aspects, treeline elevation in the Southern Alps was highest in the warmest northern exposition, intermediate in western and eastern expositions, and lowest for southern slopes. While in the Apennines treeline elevation was similar between northern, western, and eastern exposition, in contrast to the warmest southern one (Figure S2).

**FIGURE 3:**
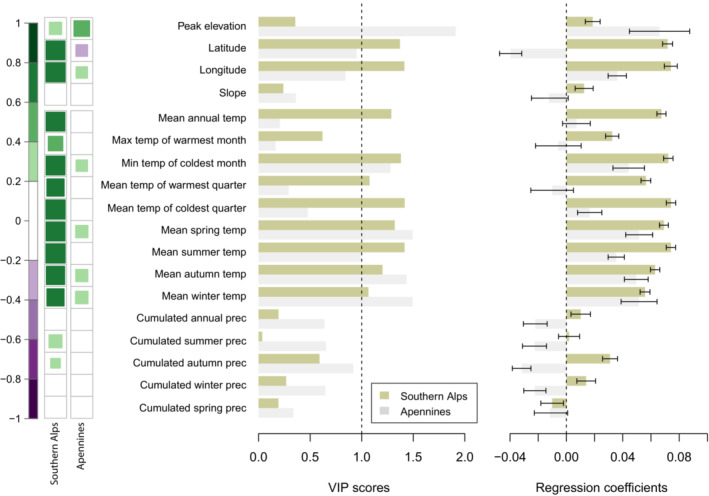
On left, a comparison of significant correlations between the southern Alps and Apennine's average treeline elevation with geographic, topographic, and climatic variables based on Spearman's rank correlation coefficient (*r*) (*p* < .05). No or negligible correlations, i.e., |*ρ*| < 0.2 are not shown. Temperature variables are scaled at sea level. Middle barplot displays the VIP (variable importance for the projection) values of first component for each explanatory variables used in the partial least squares (PLS) regressions. Explanatory variables that contribute most to the PLS model are characterized by VIPs > 1, according to Henningsson et al. (2001). Barplot on the right displays the standardized/beta coefficient test from PLS regressions and variables are not significant when confidence interval around the standardized coefficient includes zero.

**FIGURE 4:**
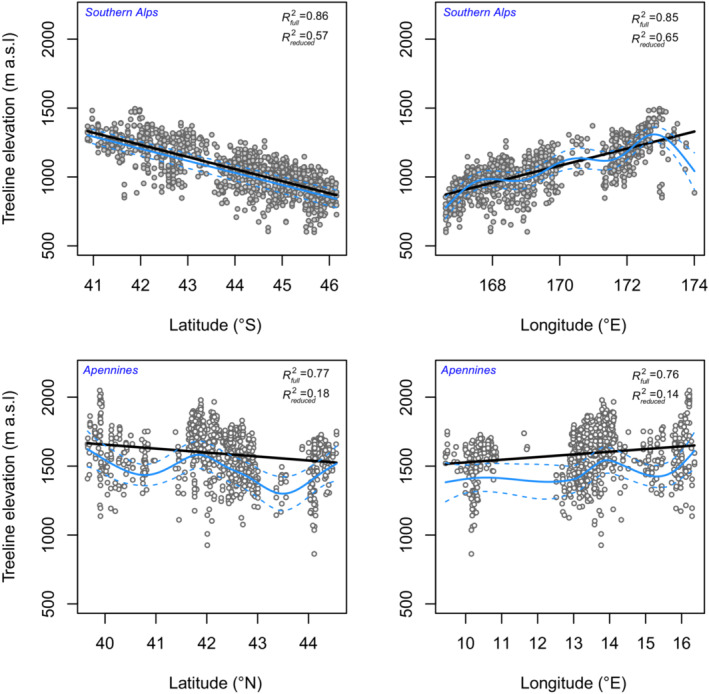
Relationship between latitude (left panels) and longitude (right panels) with treeline elevation of southern Alps and Apennines. Straight black lines represent the fitted linear mixed model while blue ones represent the fitted non‐linear generalized mixed model. Dashed lines represent 95% confidence interval of the fitted GAMM models. Values on upper right corners represent the marginal coefficient of determination of the linear mixed model.

While the results of Spearman's rank correlations and PLS regressions well overlap, the latter revealed the most important factors that control treeline elevation in the Southern Alps and Apennines by observing both the importance of variables (VIP) metric and the regression coefficients. In the PLS model of the Southern Alps, the average treeline elevation was mainly explained by the first component (~49%) and to a lesser extent by the second one (9%). For the Apennines, the first component explains 13% of variance and the second component cumulatively explains ~30% of variance. In contrast with the Apennines, treeline elevation of the Southern Alps was significantly more related to geographical variables, where latitude and longitude showed high importance (VIPs > 1, Figure 3). In the Apennines, a strong positive association between the average treeline elevation and elevation of the mountain peak and a moderate one with longitude was detected, while a negative moderate correlation with latitude was found (Figures 3 and 4). Interestingly, we found the relationship between latitude and average treeline elevation deviates from linearity only for the Apennines treelines, where the non‐linear model performs significantly better than the linear one (Figure 4). Positive relationships were observed in the Apennines between the average treeline elevation and the minimum temperature of the coldest month, and the average spring, autumn, and winter seasonal temperatures (Figure 3). In the Southern Alps, marked positive correlations were observed between treeline elevation and geo‐topographical and temperature‐associated variables, with the strongest positive correlation found with MAT, the temperature of the coldest month, the temperature of the coldest quarter, and winter temperature. Relationships with rainfall were null or fairly weak both for Apennines and Southern Alps (Figure 3). On a monthly scale, treeline elevation in the Southern Alps was positively correlated with mean temperature in all months, with the strongest correlations recorded in austral winter months, i.e., June, July, and August. In the Apennines, no significant correlations with mean monthly temperatures were found based on the Spearman's test (Figure S3). Concerning rainfall, a positive correlation with treeline elevation in the Southern Alps was found for the austral winter months, with weak and negative correlations in the summer months, i.e., January and February. In the Apennines, instead, correlations were always weak (|*ρ*| < 0.2) and not significant (Figure S3).

Although the deviance analysis has shown that nonparametric GAMM models with smoothed terms performed significantly better than parametric ones, except for latitude in the Southern Alps, only in the case of the Apennines the wiggly pattern of geographical variables strongly deviates from linearity, i.e., the linear function resides outside of the 95% confidence interval for the smooth. Furthermore, there is a sharp difference in terms of the percentage of explained deviance when comparing the fitted model with a reduced one (i.e., considering only fixed terms) for the Apennines (Figure 4, and model statistics in Table S2).

**FIGURE 5:**
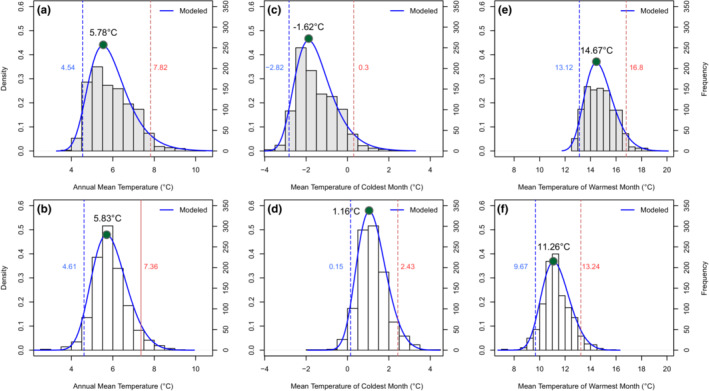
Distribution of mean annual temperature (a, b), mean temperature of the coldest month (c, d), and mean temperature of the warmest month (e, f) at treeline elevation for Apennines (upper panels, *N* = 994) and Southern Alps (lower panels, *N* = 1032). Histograms represent empirical distribution (number of breaks determined via Sturges method) while generalized extreme value (GEV) distribution is superimposed as blue lines; for each panel, left *y*‐axis is the probability (per unit on the *x*‐axis) density function for the kernel density estimation; right y‐axis is the frequency of bins; vertical blue and red dashed lines represent the 5% and 95% distribution probability, respectively.

The shape of MAT_tl_ (Figure 5, panels a,b), and the temperature of the coldest and warmest months distributions scaled at the treeline elevation (Figure 5, panels c,d and e,f, respectively), were unimodal for both mountain chains but slightly right‐skewed for MAT_tl_ in the Apennines. Results show that 95% of Apennine's' treeline occurred under MAT_tl_ spanning 4.5–7.8°C (median of all cases *μ* = 5.7°C ± 0.02), compared with 4.6 and 7.3°C (*μ* = 5.8°C ± 0.05) in the Southern Alps. In the Southern Alps, 95% of the treeline occur where the temperature of the coldest month ranges between 0.1°C and +2.4°C (*μ* = 1.1°C ± 0.04), compared with between −2.8 and +0.3 in the Apennines (*μ* = −1.6°C ± 0.06). Interestingly, no treeline occurred in the Southern Alps below −1.5°C, against ~55% of the cases in the Apennines. Concerning the temperature of the warmest month, no treelines occur in the Southern Alps above +15.6°C, with the 95% of the treelines occurring where the temperature of the warmest month ranged between +9.6°C and +13.2°C (*μ* = 11.2°C ± 0.07). In the Apennines, 95% of the treelines occurred at warmer temperatures, with warmest month temperatures between 13.1 and 16.8°C (*μ* = 14.6°C ± 0.08).

## 4 DISCUSSION

Detecting the linkage between climatic variables and the upper elevation treeline is of fundamental importance to predict the future responses of subalpine ecosystems to ongoing climate change. Although the average air temperature and treeline elevation are known to be associated, their mechanistic link is affected by the effect of other site‐specific variables, which have a different impact depending on both topographical context and historical land‐use legacy. Our approach allowed consideration of the relationship of treeline elevation with several environmental variables across two regions with contrasting historical land‐use change legacies. While it is hard to partition the effect of the topography from the macroclimate on treeline elevation, we interpreted our results with respect to the: (i) striking divergent patterns of treeline position between pole‐ and equator‐facing slopes for Apennines and Southern Alps, (ii) differential macroclimatic control on treelines elevation because of the influence of non‐climatic anthropogenic drivers, and (iii) strong isothermal difference between the treelines concerning the temperatures of the hottest/coldest month on which the two taxonomic groups grow.

### 4.1 Apennines' treelines are lower than southern Alps' treelines

The lower treeline in the Southern Alps likely reflects regional climate that is colder in New Zealand compared to the Italian Apennines, corresponding to a 2.8°C difference in the mean annual temperature scaled at sea level. Notably, the major difference between the Southern Alps and Apennines treelines limit is associated with their aspects. We found the Southern Alps treeline elevation was generally lower in shaded, i.e., colder aspects of the mountain peak, consistent with Wardle (2008) and the general expectation (Körner, 2012). Here, we quantified a 78 m offset between the treeline elevations on slopes of opposed aspects, which is an only slightly smaller offset than associated with a one degree‐step of latitude (approx. 87 m). This outcome is likely linked to the amount of solar radiation reaching the earth's surface, which sets the general thermal requirement of tree species, albeit with local‐scale topographic variations (Case & Buckley, 2015) and canopy buffering effects for the *F. sylvatica* krummholz (Rita et al., 2021). At the same elevation, lower latitude or equator‐facing forests get more solar radiation per unit area than higher latitude or polar‐facing forests, causing treeline elevation to fall with latitude and cold (but see the below discussion on the thermal limit of the species).

In contrast to the expectations for a temperature‐limited treeline (i.e., treeline elevation is higher on warmer equator‐facing slopes), the elevation of *F. sylvatica* treelines in the Apennines was found to be higher on colder, north‐facing slopes than on warmer, southern mountainsides (~114 m offset; see also Bonanomi et al., 2020). A rather different pattern in Europe has long been recognized for a limited number of Pyrenean and Alps mountains by Heybrock (1934), who found that on average, treeline was 348 m striking lower on north‐facing than on south‐facing slopes because of site‐specific topographical and microclimatic features. The most likely explanation of the low *F. sylvatica* treeline at the southern exposure (Figure 2) in the Apennines lies in the complex interaction between climate (e.g., freezing temperature, short growing season, and summer drought) and past human disturbance that shaped the actual treeline over the centuries, which goes beyond the differences imposed by the slope exposure itself. To date, concrete evidence of anthropogenic impact on treeline elevation along the Apennine chain emerged (Benatti et al., 2019; Brown et al., 2013; Compostella & Caccianiga, 2017). In the subalpine belt, centuries of logging and coppicing promoted vegetative reproduction from stump sprouting rather than clonal layering from basal branches (Saulino et al., 2022). Such an altered forest structure may generate local microclimatic feedback considerably different from those of unmanaged closed forests (Jones et al., 1997; Rita et al., 2021; Wilson & Agnew, 1992). The buffering reduction could have had long‐term effects on the light and soil moisture regime, and air temperature range enhancing the risk of photoinhibition and desiccation of *F. sylvatica* due to summer extremes (Germino & Smith, 1999), particularly in the southern/warm slopes because of the greater incident solar radiation load. Indeed, several intense summer drought spells (e.g., 2000, 2003, 2011, and 2017) caused extensive forest defoliation at the treeline across the whole Apennines (Rita et al., 2021). Several climate‐growth relationships that concerned beech forests far below their altitudinal limit (e.g., Arnič et al., 2021; Rita et al., 2014, but see Calderaro et al., 2020) reported that the radial growth of the species is strongly limited by the summer water deficiency mostly in the drought‐prone Mediterranean environments. Furthermore, open‐field at the treeline are more prone to short but intense summer drought spells (Gieger & Leuschner, 2004; Lloyd & Graumlich, 1997; Piper et al., 2016), thereby impairing forest regeneration and promoting the establishment of grasslands. Limited soil water availability would also impair *F. sylvatica* regeneration in alpine grassland, which restrains the probability of reestablishment only inside the canopy of shrub nursing species, e.g., mountain junipers and pines (Allegrezza et al., 2016; Compostella & Caccianiga, 2017). Without “nursing effect”, we expect the impact of summer drought and late frost damage would be more critical for *F. sylvatica* at the treeline, where the growing season starts later with a delay of more than 1 month at 2000 m a.s.l. in Apennine, compared to low elevations, i.e., 1000 m a.s.l. (Allevato et al., 2019; Bonanomi et al., 2021).

Other topographic factors not considered in our analysis might affect treeline elevation mostly in the Southern Alps: these include the distance from the oceanic coastline and the influence of the mountain mass (‘Massenerhebungeffekt’; Wardle, 1974). Large and tall mountains offer greater wind shelter and heat retention resulting in higher treelines than smaller, more isolated mountains.

### 4.2 Relationship between regional climate and treeline elevation

The consistency of the positive relationships between treeline elevation and temperature‐related indices of Southern Alps than Apennines highlights the importance of the climatic driver behind the *Nothofagus* treeline position, which is typical of climate‐limited treelines. However, untangling the effect of climate on treeline elevation from the effect of topography and geography can be a very complicated exercise. Earlier modeling approaches of the influence of abiotic factors on *Nothofagus* treeline position suggested that regional thermal influences account for 82% of the variation in New Zealand, reducing to 44%–52% of variation explained by a combination of thermal, physiological stress, and disturbance‐related factors operating at finer scales (Case & Duncan, 2014).

Although a clear relationship between latitudinal gradient and treeline elevations is widely recognized, mainly linked to latitude‐driven temperature variation, our results show for the Apennines a non‐linearity of the relationship between treeline elevations and geographic variables compared to a sharp pattern for the Southern Alps. In the case of the Apennines, we explain the non‐linearity between treelines elevation with latitude and longitude can be attributed to the geographical fragmentation of the distribution of high‐altitude *Fagus* forests across the Apennines, with some gaps between 41.0° and 43.5° latitudes N corresponding to lower mountain peaks, which has partly affected by greater human exploitation.

Despite the regional differences in macroclimate and despite the obvious plant functional type differences (deciduous vs. evergreen, although we recognize we have not accounted for all *Nothofagus* species commonly form the treeline in the Southern Hemisphere), our results highlight a surprising common mean annual air temperature isotherm between the *Fagus* and *Nothofagus* treelines considered here. The isotherms fall within the range of variability of the global treeline isotherm found by Körner and Paulsen (2004) and updated by Hoch and Körner (2012), as previously shown for the Southern Alps (Cieraad et al., 2014). We find a sharp climatic boundary for tree occurrences in the mountain ranges of both hemispheres. Indeed, fewer than 5% of treelines occurred where MAT_tl_ is below 4.5°C, and all treelines were found where MAT_tl_ is above 2°C.

While the position of the *Fagus* and *Nothofagus* treelines converge on similar isotherms of annual average temperature, there are striking differences between the isotherms of the coldest month. Concerning Southern Alps, our data provide strong support of early findings by Wardle (2008) who found, from data collected by meteorological stations at low elevation, correlated treeline position with the mean air temperature of the coldest month of 0°C. More recently, Cieraad and McGlone (2014) reported that coldest‐month air temperature measured at the treeline ranged between −0.1°C and +0.2°C in the abrupt treelines and from 1.6 to 1.9°C in the gradual treelines. Overall, these results agree with the thermal limits of broadleaved evergreen trees globally placed at −1°C (Ohsawa, 1990), which probably reflects the limited capability to withstand low winter temperature by the evergreen broadleaves and may set a rather narrow threshold in the onset of xylogenesis (e.g., Li et al., 2017). The strong correlation of treelines elevation with the air temperature of the cool growing season, also known as ‘growth limitation hypothesis’ (Körner, 1998), has led to the use of temperature isotherms in habitat suitability models that best‐predict future treeline expansion (e.g., Carlson et al., 2014; Maher et al., 2021). However, climate warming alone would not guarantee that treelines will rise (Harsch et al., 2009). Indeed, despite an increase in New Zealand's temperature by 0.9°C over the last 100 years (Ministry for the Environment, 2018) and growing season temperature at the treeline is above the expected minimum for growth, evidence supporting *Nothofagus* upward shift within the Southern Alps are very limited (Cullen et al., 2001; Wardle, 2008). While the effects of frosts on mature trees are unlikely to control the position of the New Zealand treeline (Cieraad et al., 2012), under clear winter skies, seedlings of *Nothofagus* above the treeline may experience limitations attributed to root zone competition, cold temperatures and photoinhibition (Germino & Smith, 1999; Wardle, 2008). These patterns have contributed to the theory that abrupt treelines emerge at elevations where substantial physiological pressures on seedling establishment would outweigh the influence of rising temperatures (Bader et al., 2021; Case & Buckley, 2015; Case & Duncan, 2014; Körner, 2021a, 2021b). The altitudinal limit of Apennine's treeline, on the other hand, is related to a lower coldest month isotherm compared to the Southern Alps counterpart. It is common knowledge that in contrast to the high vulnerability of young leaves to late frosts, dormant buds and cambium of *F. sylvatica* are cold harder and hence highly resistant to winter frost (Lenz et al., 2016). Earlier studies provided wide evidence of successful *F. sylvatica* acclimation at the cold margin of its distribution, where saplings well recruit beyond the cold distribution margin of adult trees (Vitasse et al., 2012).

Prominent differences between the Southern Alps and Apennines treelines were also found in the isotherm of the warmest month. For the Southern Alps, 95% of treelines occurred where the warmest month temperature ranged between 9.7 and 13.2°C, in agreement with data recorded from meteorological stations near treelines of around 10°C from 1120 to 1450 m a.s.l. (latitude 41°–45°S; Wardle, 2008) and 11.6°C isotherms for the treeline in the Craigieburn Range at 1300 m a.s.l. (43°S; Wardle, 1971). More recently very close values to 11.2°C were also found from both near‐ground data (Cieraad & McGlone, 2014) and model‐generated topoclimate (Case & Buckley, 2015). These values are in line with other alpine ecosystems and across latitudes such as mean temperature of 11.4°C for *Pinus sylvestris* L. in the Scottish Cairngorms (58°N; Grace, 1977), 11.2°C in the arctic treeline in Siberia (69°N; Malyshev, 1993), and 10–12°C for woodland‐grassland ecotones in the arctic region (D'Odorico et al., 2013). It is known that the isotherm of the warmest month systematically overestimates the temperatures during the growing season according to the biogeographical regions, and some have questioned its physiological relevance (Körner, 2021b). However, detected isotherm of the warmest month, which in the temperate regions of Northern Hemisphere roughly corresponds to the growing season, i.e., May to September average temperature (Figure S4), nonetheless provide interesting evidences for the Apennines, where 95% of treelines occurred within 13.1 and 16.8°C. In this regard, we again relate these warm isotherms of *F. sylvatica* treelines to the long history of non‐climate anthropogenic drivers that, over the centuries, affected Apennines' treeline position (Vacchiano et al., 2017), as well in other European regions (Malanson et al., 2011, and therein references). Today, these anthropogenic treelines (sensu Holtmeier & Broll, 2005) along the Apennines lie several hundred meters below the climatic potential of *F. sylvatica* (e.g., Benatti et al., 2019), as also indirectly demonstrated by Bonanomi et al. (2018, 2020). This would be the main reason why compared to the Southern Alps, the Apennine treeline shows a consistently higher isotherm (“*too warm*”) for the temperatures of the warmest month, in particular for the subalpine belt that was in the past subjected to more extensive human exploitation, i.e., peaking below 1900 m a.s.l. (Figure S4).

## 5 CONCLUSIONS

Research aiming to better understand the geographic pattern of treeline ecotone continues to gain momentum, particularly in view of the possible effects of global warming on the future growth and position of trees at treelines. Here, we aimed to systematically compare the general pattern between climate and treeline elevation of *F. sylvatica* and *Nothofagus* spp. along a wide latitudinal gradient in two mountain chains with similar topographic properties and contrasting land‐use legacies. We found that, while the position of the *F. sylvatica* and *Nothofagus* treelines converge on similar isotherms of annual average temperature, there were striking isothermal differences in both hottest and coldest month between these two types of treelines. Further, treeline elevation was higher on warmer slopes of the Southern Alps reflecting climate control, while the opposite was true in the Apennines. Such a counterintuitive finding was interpreted as a result of a complex interaction between past land‐use legacy and climate on the warmer slopes of the Apennines. Taken together our results support the hypothesis that the actual position of treeline in the Apennines and Southern Alps reflects the ecological processes driven by a combination of local climatic conditions, the topographic pattern, and human legacy. Projecting a certain isotherm considered to be the tree‐growth limiting factor into a warmer climate may be misleading for predicting upward shifts of subalpine forests in places where climate factors controlling treeline position may be confounded with those of land‐use change.

## AUTHOR CONTRIBUTIONS

**Angelo Rita:** Conceptualization (equal); data curation (lead); formal analysis (lead); writing – original draft (lead); writing – review and editing (equal). **Antonio Saracino:** Supervision (equal); writing – review and editing (equal). **Ellen Cieraad:** Writing – review and editing (equal). **Luigi Saulino:** Writing – review and editing (equal). **Maurizio Zotti:** Data curation (equal). **Mohamed Idbella:** Data curation (equal). **Carlo De Stefano:** Data curation (equal). **Valentina Mogavero:** Data curation (equal). **Emilia Allevato:** Data curation (equal). **Giuliano Bonanomi:** Data curation (equal); supervision (equal); writing – review and editing (equal).

## CONFLICT OF INTEREST

The authors declare they do not have any conflict of interest.

## Supporting information


Supinfo

## Data Availability

The dataset used in this study is publicly available in Figshare Digital Repository (https://doi.org/10.6084/m9.figshare.c.4836246.v1).
