# Supplementary material for: Topoclimate effect on treeline elevation depends on the regional framework: A contrast between Southern Alps (New Zealand) and Apennines (Italy) forests
Source: Ecol Evol. 2023 Jan 16;13(1):e9733. doi: 10.1002/ece3.9733 (PMC9843241; doi:10.1002/ece3.9733)
Supplement: Supplementary file 1 — Supinfo [file ECE3-13-e9733-s001.docx]

Supplementary Materials

Topoclimate effect on treeline elevation depends on the regional framework: a contrast between Southern Alps (New Zealand) and Apennines (Italy) forests.

**Authors:**

Angelo Rita^*,1^, Antonio Saracino^1^, Ellen Cieraad^2^, Luigi Saulino^1^, Maurizio Zotti^1^, Mohamed Idbella^1,3^, Carlo De Stefano^1^, Valentina Mogavero^1^, Emilia Allevato^1^, Giuliano Bonanomi^1,4^

**Affiliations:**

*1 Dipartimento di Agraria, Università di Napoli Federico II, via Università 100, 80055 Portici (NA), Italy*

*2 Research & Innovation Centre, Nelson Marlborough Institute of Technology, Nelson, New Zealand*

*3 Laboratory of Biosciences, Faculty of Sciences and Techniques, Hassan II university, Casablanca, Morocco*

*4 Task Force on Microbiome Studies, University of Naples Federico II, Naples, Italy*

**Corresponding author**: Angelo Rita; mail: angelo.unina.it; tel. +39 081 2539379

**Table S1.** Variables accounted for the analysis of treeline elevation across the Apennines and Southern Alps Mountain ranges.

| **Variable** | **Unit** |
| --- | --- |
| *Geographical* |  |
| Peak elevation | m a.s.l. |
| Latitude  Longitude | degree  degree |
| Slope | % |
| Aspect | ° |
| *Climatic* |  |
| Annual Mean Temperature (MAT)  Maximum Temperature of Warmest Month  Minimum Temperature of Coldest Month  Mean Temperature of Warmest Quarter  Mean Temperature of Coldest Quarter  Spring Mean Temperature  Summer Mean Temperature  Autumn Mean Temperature  Winter Mean Temperature  Annual Precipitation  Spring Precipitation  Summer Precipitation  Autumn Precipitation  Winter Precipitation | °C  °C  °C  °C  °C  °C  °C  °C  °C  mm y^-1^  mm season^-1^  mm season^-1^  mm season^-1^  mm season^-1^ |

‡ Slope measured across the treeline.

^*^ Monthly mean temperature from January to December.

**Table S2.** Statistics of GAMM models for Southern Alps (Tabs. *A* and *C*) and Apennines (Tabs. *B* and *D*). The approximate significance of smooth terms (s) is reported, with degree of freedoms (df), F statistics and p values.

| A. parametric coefficients | Estimate | Std. Error | t-value | p-value |
| --- | --- | --- | --- | --- |
| (Intercept) | 1048.5446 | 17.6019 | 59.5700 | *<* 0.0001 |
| B. smooth terms | edf | Ref.df | F-value | p-value |
| s(LatS) | 1.0002 | 1.0002 | 511.8699 | *<* 0.0001 |
| s(id) | 266.3082 | 304.0000 | 7.6335 | *<* 0.0001 |
| s(esposizione) | 2.9523 | 3.0000 | 82.2320 | *<* 0.0001 |
| s(slope across) | 0.7865 | 1.0000 | 19.8137 | 0.0307 |

Table A: Southern Alps

| A. parametric coefficients | Estimate | Std. Error | t-value | p-value |
| --- | --- | --- | --- | --- |
| (Intercept) | 1627.3912 | 26.9577 | 60.3683 | *<* 0.0001 |
| B. smooth terms | edf | Ref.df | F-value | p-value |
| s(Lat.N) | 6.5783 | 6.7107 | 11.6701 | *<* 0.0001 |
| s(Picco) | 259.8829 | 299.0000 | 9.3214 | *<* 0.0001 |
| s(esposizione) | 2.9633 | 3.0000 | 112.6940 | *<* 0.0001 |
| s(slope across) | 0.9441 | 1.0000 | 125.7190 | *<* 0.0001 |

Table B: Apennines

| A. parametric coefficients | Estimate | Std. Error | t-value | p-value |
| --- | --- | --- | --- | --- |
| (Intercept) | 1045.1220 | 17.5155 | 59.6683 | *<* 0.0001 |
| B. smooth terms | edf | Ref.df | F-value | p-value |
| s(LongE) | 7.9428 | 8.0866 | 95.8648 | *<* 0.0001 |
| s(id) | 248.7477 | 304.0000 | 5.1384 | *<* 0.0001 |
| s(esposizione) | 2.9531 | 3.0000 | 78.0251 | *<* 0.0001 |
| s(slope across) | 0.8119 | 1.0000 | 16.9188 | 0.0214 |

Table C: Southern Alps

| A. parametric coefficients | Estimate | Std. Error | t-value | p-value |
| --- | --- | --- | --- | --- |
| (Intercept) | 1628.4939 | 26.9942 | 60.3276 | *<* 0.0001 |
| B. smooth terms | edf | Ref.df | F-value | p-value |
| s(Long.N) | 5.9744 | 6.0636 | 7.7577 | *<* 0.0001 |
| s(Picco) | 263.3221 | 299.0000 | 10.1121 | *<* 0.0001 |
| s(esposizione) | 2.9630 | 3.0000 | 114.2571 | *<* 0.0001 |
| s(slope across) | 0.9461 | 1.0000 | 140.9460 | *<* 0.0001 |

Table D: Apennines

**Figure S1**. Example of measurements taken in Te Anau Peak (44°51’56.63’’ S; 167°55’16.96’’ E) to assess treeline elevation. For each of the 294 Southern Alps peaks, treeline contours were traced as a linear feature digitizing them all along the mountain aspects. For each aspect of the digitized contour lines maximum, minimum and average elevation above sea level was assessed.


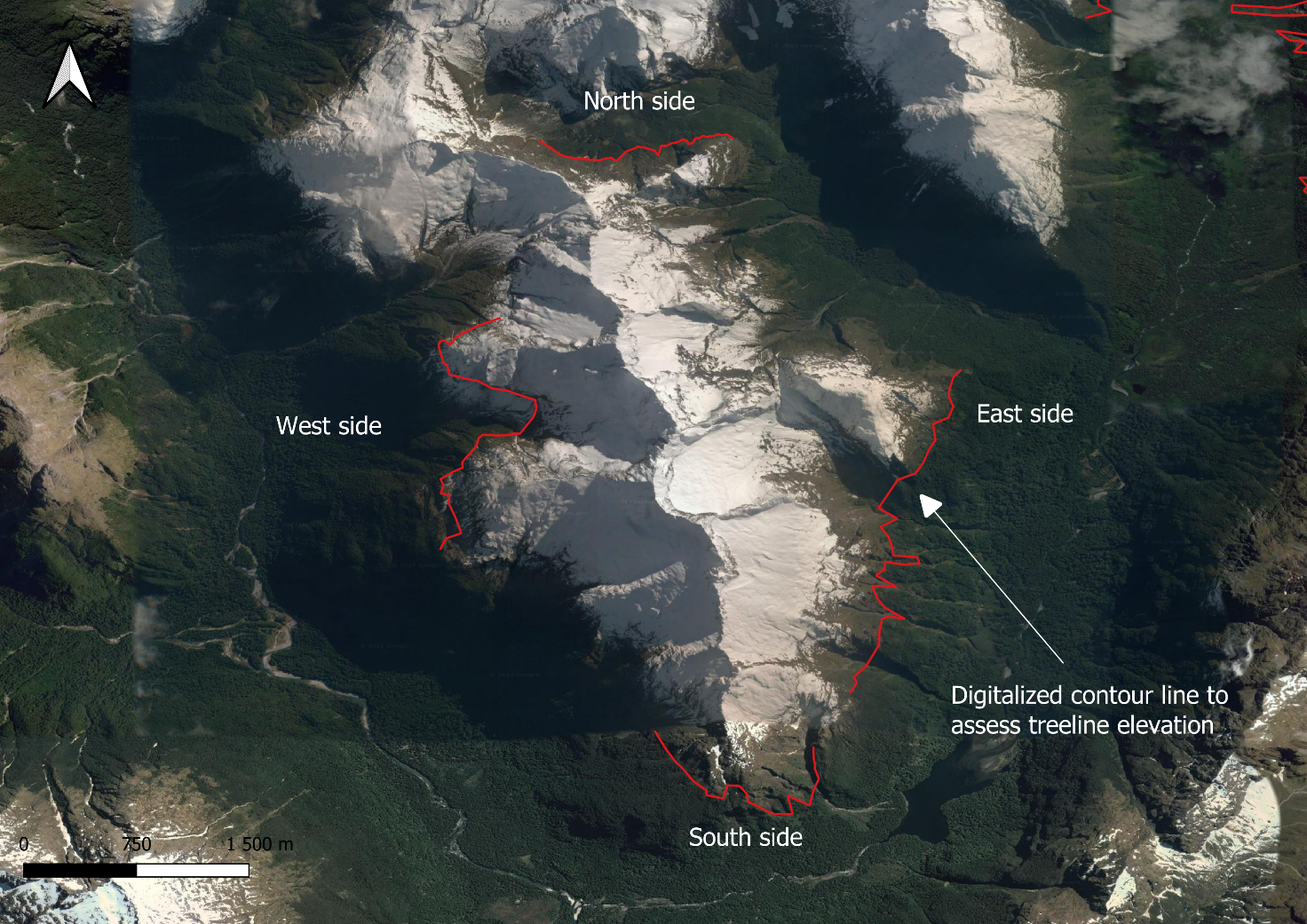


**Figure S2**. Average treeline elevation in the Southern Alps (*a*) and Apennines (*b*) according to the aspect: each box represents the median with respective quartiles; upper and lower whiskers reach the largest and smallest observations.


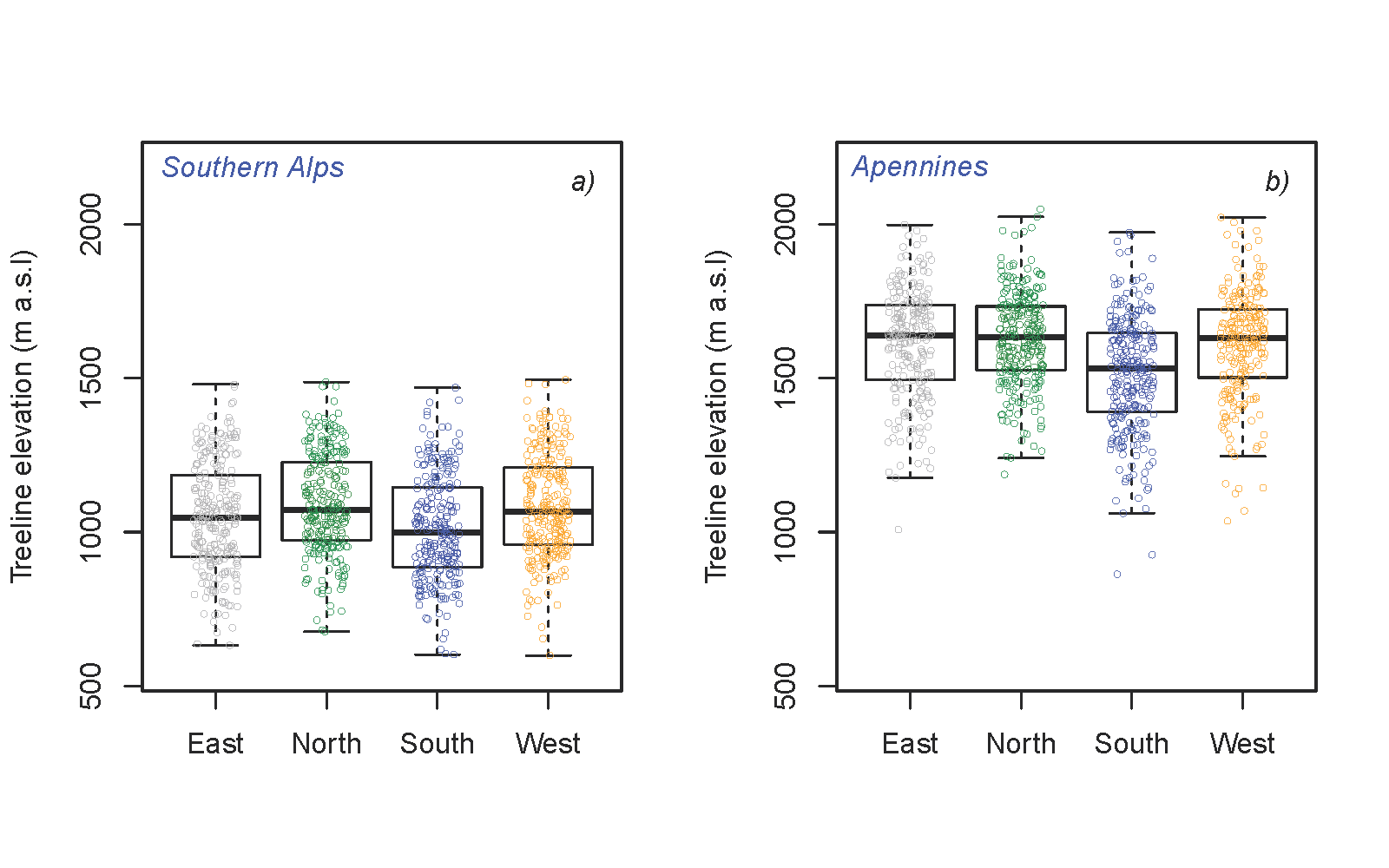


**Figure S3**. Correlation (Spearman rho coefficient) between treeline elevation and mean monthly temperature adjusted at sea level (MMT_adj_) (panel *a*) and monthly precipitation (panel *b*) in the Southern Alps (square) and Apennines (circles).


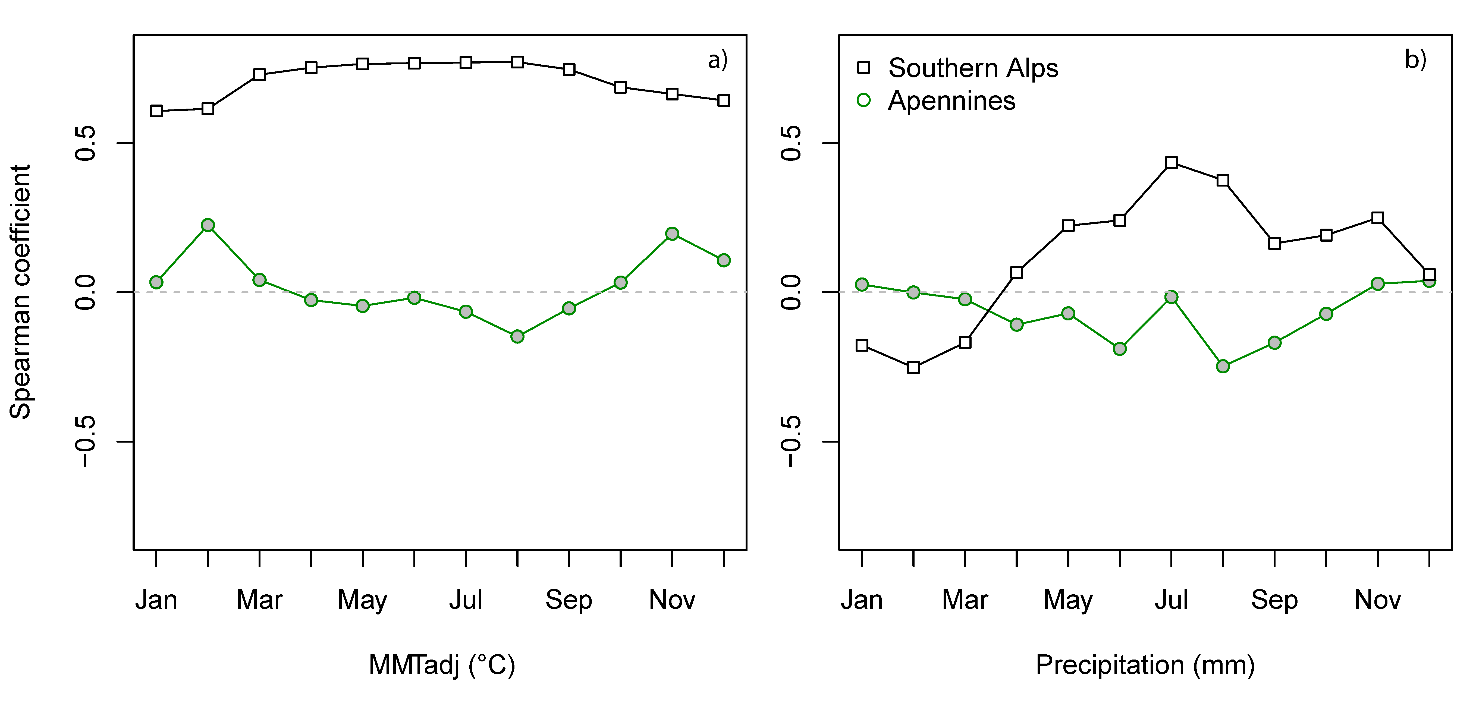


**Figure S4**. Distribution of mean temperature of the Northern Hemisphere growing season (from May to September) at treeline elevation for Apennines (upper panels, N=994) for the whole dataset. The histogram in the left panel represents the empirical distribution while the Generalized Extreme Value (GEV) distribution is superimposed as blue dashed line; vertical lines represent the 5% and 95% distribution probability in blue and red, respectively. Right panel shows the GEV distribution of the mean temperature of the growing season (from May to September) for Apennines’ mountains with peaks below and above 1900 m a.s.l..

**
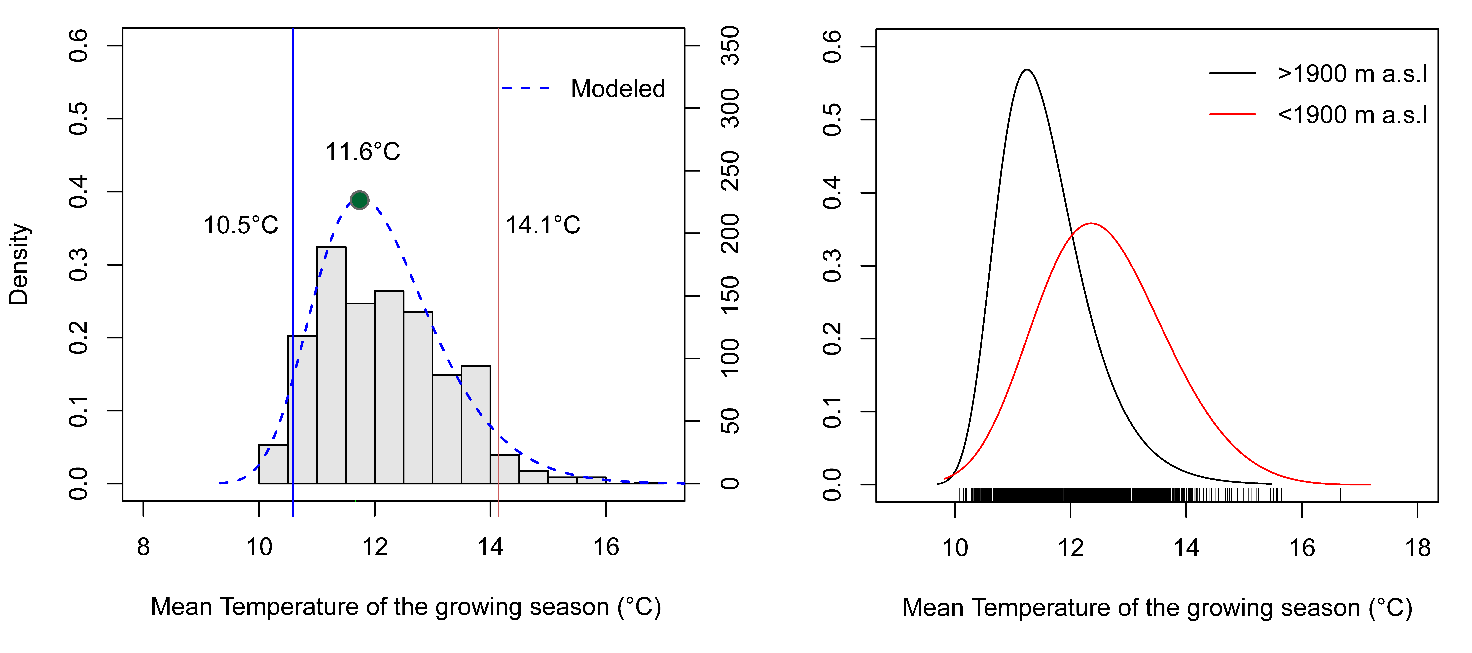
**
